# Early-Life Disadvantages and Late-Life Cardiometabolic Health Among Older Adults in Brazil

**DOI:** 10.1093/geroni/igaf122.3420

**Published:** 2025-12-31

**Authors:** Brenda Marques, Mateo Farina

**Affiliations:** The University of Texas at Austin, Austin, Texas, United States; University of Texas at Austin, Austin, Texas, United States

## Abstract

Exposures to early-life disadvantages, including low parental socioeconomic status (SES), have been linked to long-term health consequences through multiple physiological and social pathways, contributing to higher mortality risk and increased cardiometabolic dysfunction. However, research on these associations remains limited, particularly in low- and middle-income countries. Hence, research on the long-term effects of early-life SES on cardiometabolic risk factors in late adulthood remains scarce and nevertheless needed, with some works calling for attention to the role of socioeconomic-related childhood adversities in cardiometabolic health among Brazilian adults (Andrade et al., 2016). Using data from the 2015 wave of the Brazilian Longitudinal Study of Aging (ELSI-Brazil), this study examines one source of early-life disadvantage and its association with cardiometabolic health among 1408 older Brazilian adults. We have applied generalized linear regression models for continuous metabolic health parameters and logistic regression models for categorical outcomes. Preliminary findings are mixed: on the one hand, having parents with at least some education is protective against stroke risk in comparison to individuals whose parents never went to school and individuals whose parents had bachelor’s degree. When it comes to diabetes, individuals whose parents had bachelor’s degree or higher education attained have 43.7% higher odds of diabetes compared to individuals whose parents never went to school.

